# Inter-Trial Variability in Sensory-Evoked Cortical Hemodynamic Responses: The Role of the Magnitude of Pre-Stimulus Fluctuations

**DOI:** 10.3389/fnene.2012.00010

**Published:** 2012-11-05

**Authors:** Mohamad Saka, Jason Berwick, Myles Jones

**Affiliations:** ^1^Animal Imaging Service Unit, King Fahad Centre for Medical Research, King Abdulaziz UniversityJeddah, Saudi Arabia; ^2^Department of Psychology, The Centre for Signal Processing in NeuroImaging and Systems Neuroscience, University of SheffieldSheffield, UK

**Keywords:** intertrial variability, cortical hemodynamics, optical imaging, barrel cortex, spontaneous fluctuation

## Abstract

Brain imaging techniques utilize hemodynamic changes that accompany brain activation. However, stimulus-evoked hemodynamic responses display considerable inter-trial variability and the sources of this variability are poorly understood. One of the sources of this response variation could be ongoing spontaneous hemodynamic fluctuations. We recently investigated this issue by measuring cortical hemodynamics in response to sensory stimuli in anesthetized rodents using 2-dimensional optical imaging spectroscopy. We suggested that sensory-evoked cortical hemodynamics displayed distinctive response characteristics and magnitudes depending on the phase of ongoing fluctuations at stimulus onset due to a linear superposition of evoked and ongoing hemodynamics (Saka et al., 2010). However, the previous analysis neglected to examine the possible influence of variability of the size of ongoing fluctuations. Consequently, data were further analyzed to examine whether the size of pre-stimulus hemodynamic fluctuations also influenced the magnitude of subsequent stimulus-evoked responses. Indeed, in the case of all individual trials, a moderate correlation between the size of the pre-stimulus fluctuations and the magnitudes of the subsequent sensory-evoked responses were observed. However, different correlations between the size of the pre-stimulus fluctuations and magnitudes of the subsequent sensory-evoked cortical hemodynamic responses could be observed depending on their phase at stimulus onset. These analyses suggest that both the size and phase of pre-stimulus fluctuations in cortical hemodynamics contribute to inter-trial variability in sensory-evoked responses.

## Introduction

The changes in blood flow, volume, and oxygenation that accompany brain activation are collectively referred to as the hemodynamic response. Cerebral hemodynamics are of interest to cognitive neuroscience as they form the basis of non-invasive human brain imaging techniques such as Blood Oxygenation Level Dependent (BOLD) fMRI (Kwong et al., 1992; Ogawa et al., 1992). However, stimulus-evoked hemodynamic responses are known to display considerable inter-trial variability (Aguirre et al., 1998); and the sources of this variation are poorly understood. The ubiquitous low frequency fluctuations in “resting state” hemodynamics (Biswal et al., 1995; Mayhew et al., 1996; Lowe et al., 1998; Obrig et al., 2000; Spitzer et al., 2001; Greicius et al., 2003; Majeed et al., 2009) could be one possible source of the variation in subsequent stimulus-evoked responses. In the case of ongoing and evoked hemodynamics, a recent study suggests that ongoing hemodynamic activity may contribute to the variation in stimulus-evoked hemodynamic responses (Fox et al., 2006) in a similar fashion to that in which resting cortical activity contributes to the variance in evoked neural responses (Arieli et al., 1996). Fox and colleagues measured task related BOLD signal changes in motor cortex and found inter-trial variability. Subsequent subtraction of coherent spontaneous BOLD fluctuations from the cortex contralateral to that activated by the task, reduced inter-trial variability and therefore suggested a superposition of evoked and ongoing hemodynamics. We previously investigated this phenomenon in a well defined animal model, the whisker barrel somatosensory cortex of the rodent (Saka et al., 2010). This animal model allowed the use of invasive 2-dimensional optical imaging spectroscopy (2D-OIS, Devor et al., 2003; Berwick et al., 2005, 2008) that can measure the individual components of the hemodynamic response (oxyhemoglobin, deoxyhemoglobin, and total hemoglobin) at a higher temporal resolution than that afforded by fMRI. Presentation of sensory stimuli to the whiskers elicited hemodynamic responses in the somatosensory cortex that could be examined on a trial by trial basis. Individual trials were then averaged according to the phase of their pre-stimulus hemodynamic fluctuations. This revealed that cortical hemodynamics display distinct responses to sensory stimuli depending on the phase of pre-stimulus fluctuations (Figure 4). As in the somatosensory system of the anesthetized rodent sensory stimuli have been shown to elicit changes in metabolic and hemodynamic parameters in cortex both ipsilateral and contralateral to the sensory stimulus (Devor et al., 2008; Boorman et al., 2010) adopting a similar approach to Fox and colleagues in this animal model would have resulted in the subtraction of sensory “responses” rather than just coherent spontaneous fluctuations. Therefore, to investigate the origin of the difference in response depending on phase, null trials of identical duration were collected where no stimuli were presented. Phase averaged null trials were subtracted from their phase averaged counterparts and the similarity of the resultant time series strongly suggested that the mechanism that resulted in the difference between phase averaged trials was a linear superposition of ongoing and evoked cortical hemodynamics (Figure 4). However, this previous analysis neglected to examine the possible influence of the size of the pre-stimulus hemodynamic fluctuations. Indeed, if our previous assertion regarding a linear superposition was correct then the size of the pre-stimulus fluctuation should also influence hemodynamic response magnitude. Thus to further examine inter-trial variability in sensory-evoked hemodynamic responses in terms of ongoing pre-stimulus fluctuations, subsequent analyses are performed to examine the putative influence of the inter-trial variability in size of pre-stimulus fluctuation and the magnitude of subsequent evoked responses.

## Materials and Methods

As data has been previously presented (Saka et al., 2010) the experimental methods are explained only briefly here.

### Animal preparation (*n* = 6)

Female Hooded Lister rats weighing between 250 and 400 g were kept in a 12 h dark/light cycle environment at a temperature of 22°C with food and water *ad libitum*. Prior to surgery, animals were anesthetized with urethane (1.25 g/kg i.p.). Rectal temperature was maintained at 37°C throughout surgical and experimental procedures using a homeothermic blanket (Harvard). Animals were tracheotomized to allow artificial ventilation and measurement of end-tidal CO_2_. Ventilation parameters were adjusted to maintain blood gas measurements and end-tidal CO_2_ within physiological limits. The femoral vein and artery were cannulated to allow drug infusion and measurement of mean arterial blood pressure respectively. Phenylephrine (0.13–0.26 mg/h) was infused to maintain blood pressure between physiological limits (MABP, 100–110 mmHg). Animals were placed in a stereotaxic frame (Kopf Instruments) and the skull overlying the somatosensory cortex was thinned to translucency with a dental drill under constant cooling with saline. A plastic “well” was attached to the thinned skull and filled with saline (37°C) to reduce specularities from the skull surface. All procedures were carried out in accord with Home Office regulations.

### 2D optical imaging spectroscopy estimates of hemoglobin changes

Images of the cortical surface were collected with a high-speed CCD camera. The cortex was sequentially illuminated with four wavelengths (two pairs) of light (495 ± 31 and 587 ± 9 nm FWHM; 559 ± 16 and 575 ± 14 nm FWHM) with a Lambda DG-4 high-speed filter changer (Sutter Instrument, Novato, CA, USA) and stabilized 300W xenon arc light source. The wavelengths in each pair are chosen such that they sample a similar tissue volume. However, for each of the two wavelengths in each pair, one is associated with a greater absorption co-efficient for oxyhemoglobin than deoxyhemoglobin and the other is associated with a greater absorption co-efficient for deoxyhemoglobin than oxyhemoglobin. The camera data collection (30 Hz) was synchronized to filter changing such that each subsequent image was collected with a different wavelength of cortical illumination. This “multi-wavelength” optical imaging data were subject to spectral analysis using a modified Beer Lambert law that corrects for the wavelength dependency of photon path length (Mayhew et al., 1999) and has been used previously to analyze this form of spectroscopic data (Berwick et al., 2005, 2008). This permitted an estimation of changes in total hemoglobin concentration (Hbt), oxyhemoglobin concentration (HbO_2_), and deoxyhemoglobin concentration (Hbr). As data from each of the four wavelengths of illumination was required for spectral estimates of hemoglobin changes, the effective sampling frequency was that of the camera frame rate divided by 4 (30/4 = 7.5). The baseline value of cortical hemoglobin concentration was set at 104 μM which was estimated by a previous MRI study in rodent (Kennerley et al., 2005).

### Stimulus presentation, paradigms, and data analysis

All stimulus presentation was controlled through a 1401plus (CED Ltd, UK) running custom-written code with stimulus onset time locked to the CCD camera. Electrical stimulation of the whole whisker pad was delivered via stainless steel electrodes inserted in an anterior direction each side of the whisker pad (Mayhew et al., 2000; Jones et al., 2001, 2002, 2004, 2005; Sheth et al., 2003). All electrical stimuli were presented for 3 s at 1 Hz with a 0.3 ms individual pulse width at an intensity of 1.2 mA (Jones et al., 2004, 2005, 2008). No changes in MABP, heart rate, or PCO_2_ were observed at this stimulus intensity suggesting that the measured hemodynamic response was not contaminated by systemic physiological changes. Trials were 24 s long with a 1 s inter-trial interval. Stimulation occurred on the eight second of each trial. Experimental runs consisted of 30 trials. In each experimental run stimuli were presented or data with identical parameters were collected without presenting stimuli. Six to nine experimental runs (180–270 trials) where stimuli were presented (termed “stimulus-evoked”) were collected for each animal (*n* = 6).

For each animal, trial-averaged images of total hemoglobin concentration changes following stimulus presentation were analyzed using a signal source separation algorithm (Molgedey and Schuster, 1994) as previously described (Zheng et al., 2001). This procedure has been shown on numerous occasions to localize spatially discrete activations of barrel cortex which show excellent concordance with cytochrome oxidase histology in tangential (Jones et al., 2001, 2002) and coronal sections (Jones et al., 2004). “Barrel maps” were registered with images of cortical surface for selection of a region of interest (ROI). A parenchymal ROI was selected in the center of the active barrel region avoiding overlying surface vasculature. This ROI was used to provide time series of hemodynamics for each “stimulus-evoked” trial for each animal.

## Analyses and Results

### Individual trial data display inter-trial variability and spontaneous pre-stimulus fluctuations

**Figure 1:**
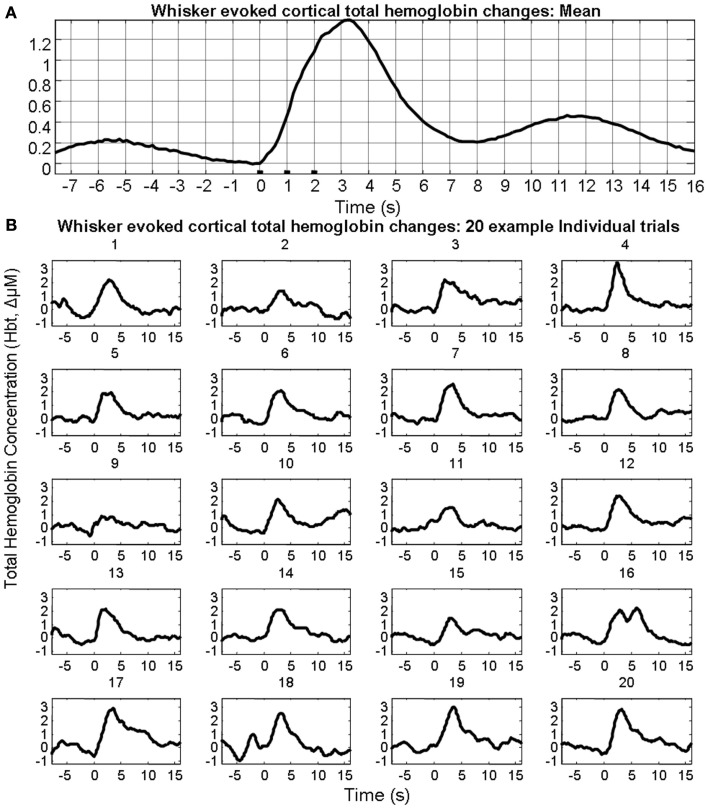
**Sensory stimuli evoked hemodynamic responses in contralateral somatosensory cortex**. **(A)** Trial and animal averaged cortical total hemoglobin response to whisker pad stimuli. **(B)** Example of 20 individual “un-averaged” trials.

In the interests of brevity and clarity analyses are shown for total hemoglobin concentration (Hbt) alone. Sensory stimuli evoked (Figure 1A) hemodynamic responses in the contralateral cortex that could be observed following trial and animal averaging. However, individual trials displayed considerable variability both in terms of evoked responses and pre-stimulus fluctuations (Figure 1B).

### Metrics of the size of pre-stimulus fluctuations and response magnitude

**Figure 2:**
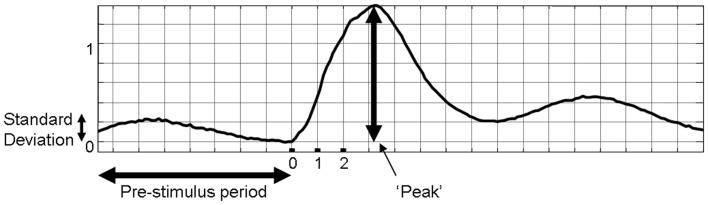
**Metrics of individual trials of sensory-evoked total hemoglobin concentration changes that were used for subsequent analyses**.

Our previous investigation demonstrated that the phase of the pre-stimulus fluctuations in cerebral hemodynamics at stimulus onset influenced the magnitude of subsequent stimulus-evoked hemodynamics (Saka et al., 2010). However, the analysis neglected to examine whether the magnitude of the pre-stimulus fluctuation influenced the magnitude of the sensory-evoked response. To investigate these issues further analyses were performed on the data set presented in (Saka et al., 2010). As the pre-stimulus fluctuations in total hemoglobin concentration are oscillatory in nature, examining the magnitude of the time series at a particular time point would not accurately characterize the size of the fluctuation. As such, the standard deviation of the fluctuation of the entire pre-stimulus time period (8 s) was chosen as the metric of the size of the pre-stimulus fluctuation. The standard deviation has the advantage of being expressed in the same units as the data (unlike variance) and is more insensitive to DC deviations from zero than the root mean square (RMS). However, similar results can be obtained regardless of which metric is chosen (variance, standard deviation, RMS, data not shown). The standard deviation of 8 s pre-stimulus of each trial was calculated as a measure of the magnitude of the pre-stimulus fluctuation (Figure 2). The peak of the response following stimulus presentation was taken as a metric of response magnitude (Figure 2).

### Association between size of pre-stimulus fluctuations and response magnitude

**Figure 3:**
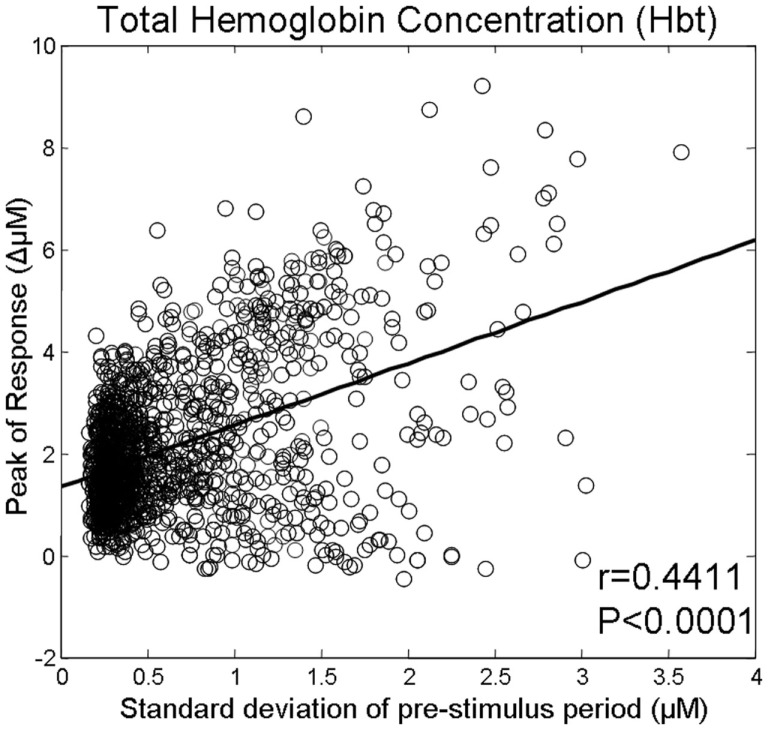
**Magnitude of whisker evoked Total Hemoglobin Concentration (Hbt) responses compared to the size of ongoing pre-stimulus fluctuations for each individual trial**.

A total of 1404 trials were taken from six animals and were then used to investigate whether the magnitude of the pre-stimulus fluctuations was related to the inter-trial variability of sensory-evoked cortical hemodynamic responses. The size of the pre-stimulus fluctuations was modestly (*R* = 0.44) but significantly (*p* < 0.0001) correlated with response magnitude (Figure 3).

### Phase of spontaneous pre-stimulus hemodynamic fluctuations at stimulus onset

**Figure 4:**
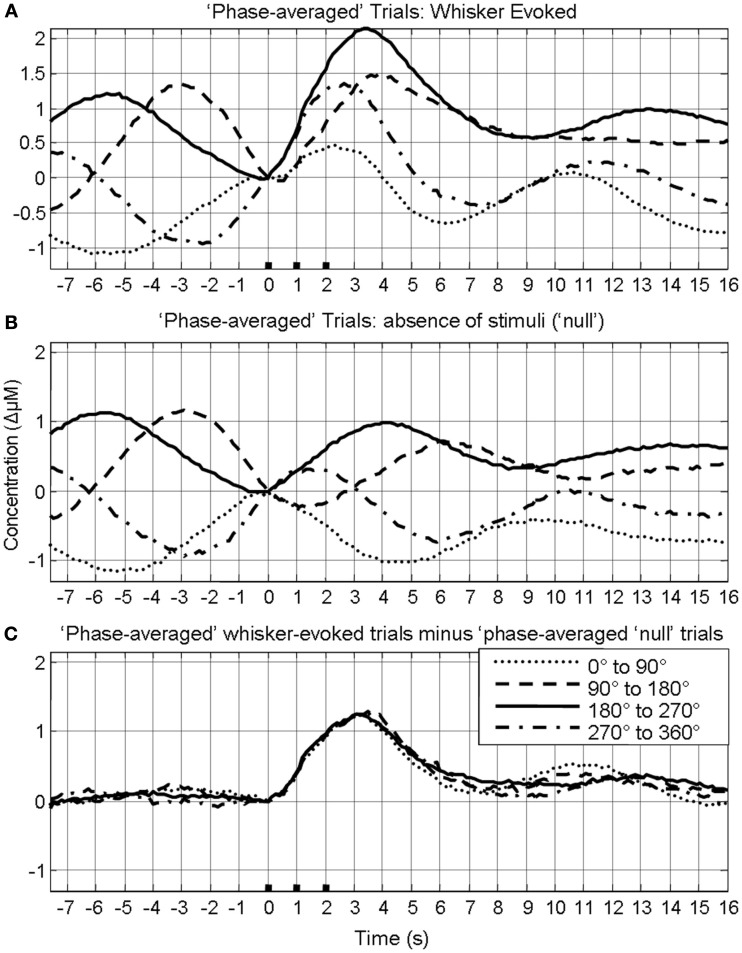
**Linear superposition of sensory-evoked and ongoing cortical total hemoglobin concentration changes (Hbt)**. **(A)** Hbt responses to sensory stimuli were grouped based on the phase of their pre-stimulus fluctuations at stimulus onset and averaged. **(B)** Hbt trial data without presentation of sensory stimuli (“nulls”) are grouped based on the phase of their pre-stimulus fluctuations at the same time point within each trial and averaged. **(C)** Subtraction of the phase averaged stimulus-evoked responses from their phase averaged null counterpart results in four similar time series.

**Figure 5:**
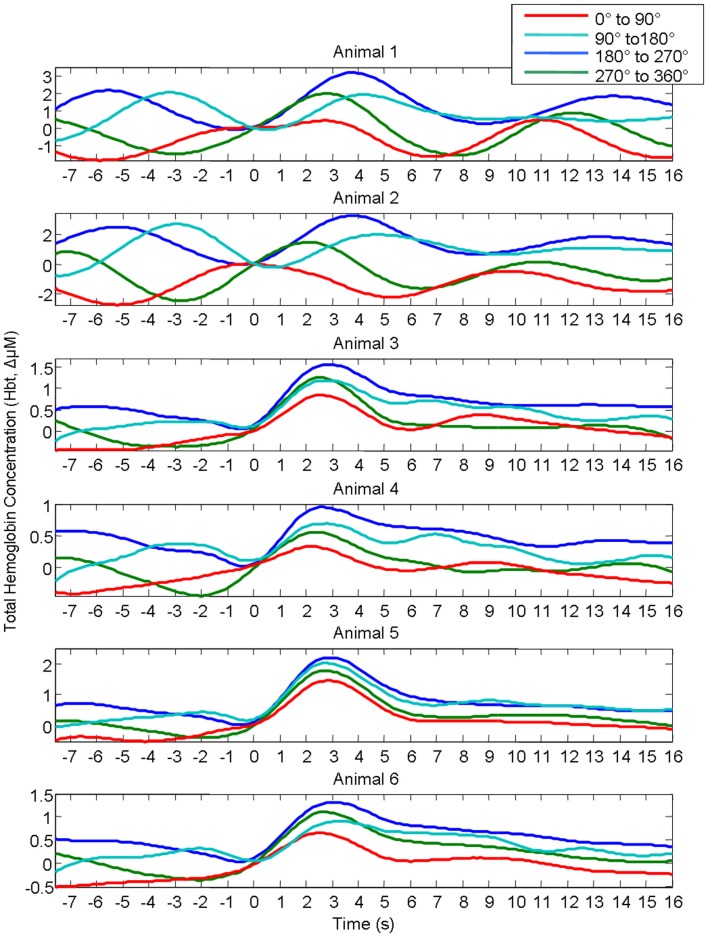
**Rather than grouping all individual trials together from all animals, Hbt responses to sensory stimuli in each individual animal were grouped based on the phase of their pre-stimulus fluctuations at stimulus onset and averaged**. In each individual animal responses differ depending on phase group.

In our previous investigation this linear superposition of ongoing and evoked hemodynamics resulted in different evoked response magnitude because depending on the phase of the ongoing fluctuation at stimulus onset, the fluctuation was either rising or descending. If the ongoing fluctuation was descending at stimulus onset, the ongoing fluctuation “subtracted” from the evoked response, resulting in responses of smaller magnitude. For other phases where the ongoing fluctuations were rising, the ongoing fluctuation “added” to the response, resulting in responses of larger magnitude. This phenomenon of linear superposition of evoked and ongoing cortical hemodynamics was suggested by comparing “phase averaged” stimulus-evoked trials with trials collected in the absence of stimuli. The results of this analysis are displayed again to aid the reader. In short, trials were assigned to one of four groups (0–90°; 90–180°; 180–270°; 270–360°) based on the phase of pre-stimulus hemodynamic fluctuation at stimulus onset. A Hilbert transform (Matlab™ function “Hilbert”) was used to calculate the phase of the ongoing pre-stimulus fluctuations in total hemoglobin concentration (Le Van Quyen et al., 2001; Pikovsky et al., 2001; Haslinger et al., 2006; Le Van Quyen and Bragin, 2007; Saka et al., 2010). The magnitude of the stimulus-evoked increases in Hbt was smallest in the 0–90° group, followed by in the 270–360° group, and 90–180° groups and was largest in the 180–270° group (Figure 4). Data from trials in which no stimuli were presented were also averaged based on the phase of their Hbt fluctuations at a time point that corresponded to stimulus onset (8 s in each trial) in the stimulus presentation trials. This resulted in “phase averaged” time series that were identical to phase averaged stimulus-evoked responses during the “pre-stimulus” period (compare Figures 4A,B). However, the time series differed after stimulus presentation with the phase averaged null trials providing a useful indication of the average “behavior” of the hemodynamics for each phase group had a stimulus not occurred. To demonstrate that these differences in stimulus-evoked responses in each phase group were due to superposition of ongoing and evoked hemodynamics, phase averaged “nulls” were subtracted from “phase averaged” stimulus-evoked responses. This subtraction resulted in four similar time series that closely resembled the time series of the mean of all trials without phase averaging (Figure 4C). In the previous investigation trials were taken from all animals and grouped together based on their phase at stimulus onset (Saka et al., 2010). Before conducting subsequent analyses, the data was examined in each animal to confirm that this phenomenon was evident in each individual animal (Figure 5).

### Association between the size of pre-stimulus fluctuations and response magnitude varies depending on the phase of the pre-stimulus fluctuations at stimulus onset

**Figure 6:**
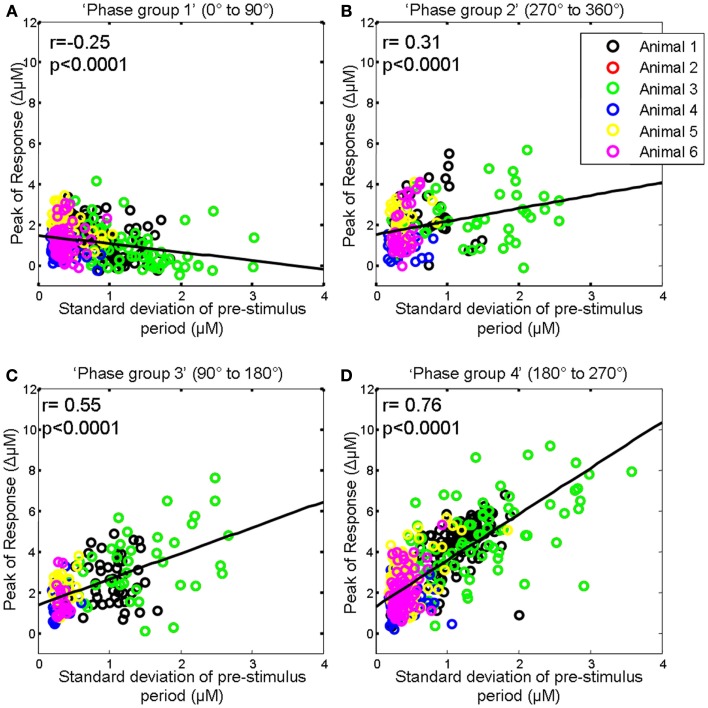
**Magnitude of whisker evoked Total Hemoglobin Concentration (Hbt) compared to the size of ongoing pre-stimulus fluctuations for each individual trial for each phase group**. **(A)** Magnitude of evoked Hbt responses compared to the size of ongoing pre-stimulus fluctuations for each individual trial in “Phase group 1” (0–90°). **(B)** Magnitude of evoked Hbt responses compared to the size of ongoing pre-stimulus fluctuations for each individual trial in “Phase group 2” (270–360°). **(C)** Magnitude of evoked Hbt responses compared to the size of ongoing pre-stimulus fluctuations for each individual trial in “Phase group 3” (90–180°). **(D)** Magnitude of evoked Hbt responses compared to the size of ongoing pre-stimulus fluctuations for each individual trial in “Phase group 4” (180–270°). Trials from each individual animal are shown as different colors.

As depending on phase, the ongoing fluctuation could effectively reduce or increase the magnitude of the evoked response (Figure 4), we therefore then investigated whether the correlation between the magnitude of the pre-stimulus hemodynamic fluctuation and the sensory-evoked hemodynamic response magnitude differed for each of the previously defined phase groups (0–90°; 90–180°; 180–270°; 270–360°; Saka et al., 2010). For ease of interpretation we display the phase groups in the order associated with increasing evoked response magnitude, i.e., group 1 (0–90°); group 2 (270–360°); group 3 (90–180°); and group 4 (180–270°) when describing the results of subsequent analyses. For trials in the first group (0–90°) the ongoing fluctuation descended at stimulus onset thus subtracting from the stimulus-evoked response resulted in the smallest evoked response (compare Figure 4B with Figure 4A). As such, in this phase group it would be expected that the magnitude of the pre-stimulus fluctuation would be negatively correlated with response size. Indeed if the magnitude of the pre-stimulus fluctuations for each trial in this phase group are examined there is a modest negative correlation (*r* = −0.25, *p* < 0.0001, Figure 6A). For trials in the fourth group (180–270°) the ongoing fluctuation “ascended” following stimulus presentation and peaked at a similar time to the evoked response (compare Figure 4B with Figure 4A). Consequently, the largest response occurred in this phase group as the “largest” addition of ongoing and evoked hemodynamics could occur (Figure 4). Indeed the correlation between the pre-stimulus fluctuation magnitude and evoked response magnitude was greatest in this phase group (Figure 6D). In the second (270–360°) and third group (90–180°) the ongoing fluctuations are positive during the time period of the evoked response although they do not peak at an identical time point to the evoked response (compare Figure 4B with Figure 4A). As such, a more modest correlation would be expected than for trials than in the fourth group (180–270°). Indeed the correlations were *r* = 0.31, *p* < 0.0001 and *r* = 0.55, *p* < 0.0001 respectively (Figures 6B,C).

### Association between the size of pre-stimulus fluctuations can be increased if the “polarity” of the size metric is altered depending on phase group

**Figure 7:**
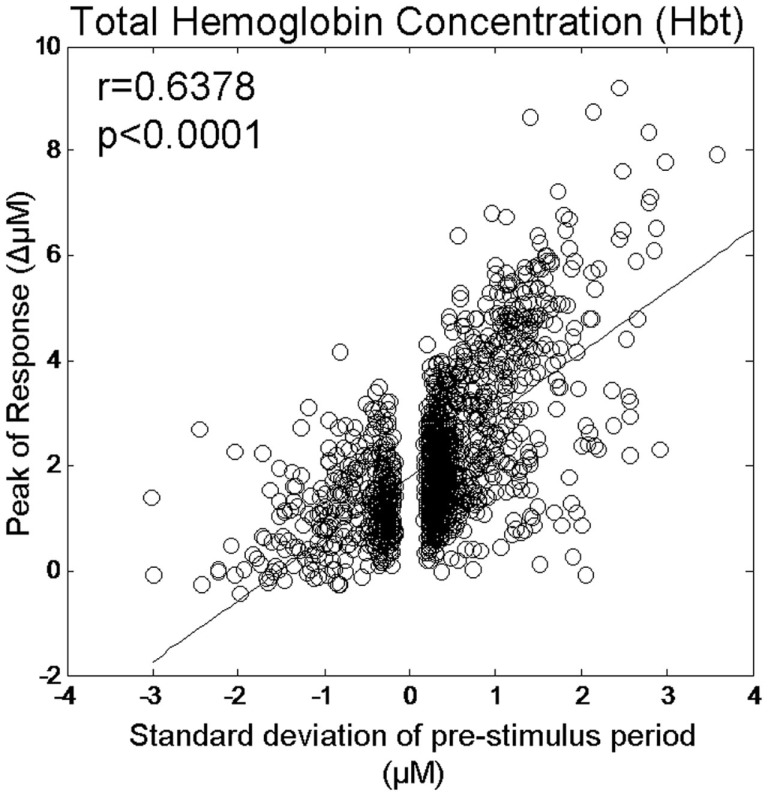
**Magnitude of evoked Hbt responses compared to the magnitude of ongoing pre-stimulus fluctuations for each individual trial following reversal of polarity of pre-stimulus values in phase group 1 (0–90°)**.

As positive correlations between hemodynamic response magnitude and the size of the pre-stimulus hemodynamics fluctuation were observed in the second (270–360°), third group (90–180°), and fourth group (180–270°) these values were left unaltered. However, as the pre-stimulus magnitudes in the first phase group (0–90°) were negative we altered the polarity (i.e., multiplied by −1) of the pre-stimulus fluctuation sizes for this group. We then re-calculated the correlation co-efficient between pre-stimulus fluctuation size and response magnitude for individual trials from all phase groups (Figure 7). This resulted in a higher correlation co-efficient (*r* = 0.64) than was observed before altering the polarity of the pre-stimulus size values in the first group (*r* = 0.44).

**Figure 8:**
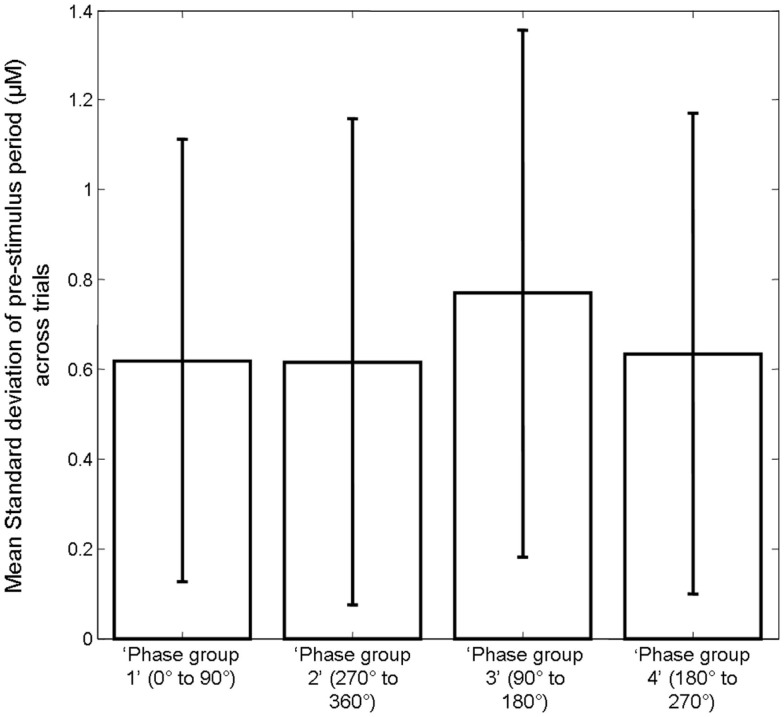
**Bar chart displaying the mean size of the pre-stimulus fluctuations in Hbt in each trial in each phase group**. Error bars are SD.

**Figure 9:**
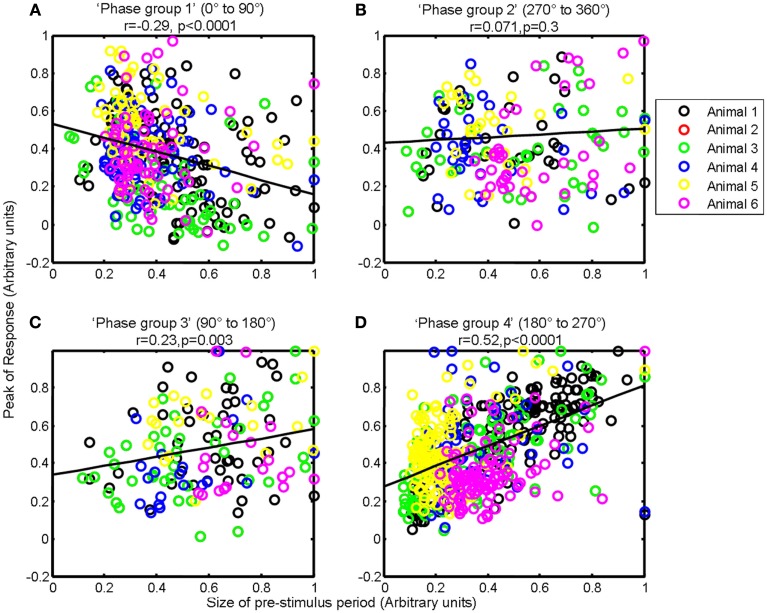
**Magnitude of whisker evoked Total Hemoglobin Concentration (Hbt) compared to the size of ongoing pre-stimulus fluctuations for each individual trial for each phase group**. For each animal, for each phase group size the sizes of ongoing pre-stimulus fluctuations and magnitudes of responses were normalized between zero and unity. **(A)** Magnitude of evoked Hbt responses compared to the size of ongoing pre-stimulus fluctuations for each individual trial in “Phase group 1” (0–90°). **(B)** Magnitude of evoked Hbt responses compared to the size of ongoing pre-stimulus fluctuations for each individual trial in “Phase group 2” (270–360°). **(C)** Magnitude of evoked Hbt responses compared to the size of ongoing pre-stimulus fluctuations for each individual trial in “Phase group 3” (90–180°). **(D)** Magnitude of evoked Hbt responses compared to the size of ongoing pre-stimulus fluctuations for each individual trial in “Phase group 4” (180–270°). Trials from each individual animal are shown as different colors.

However, observation of the scatter plots on an individual animal basis (each color corresponds to an individual animal’s data) suggests that in addition to inter-trial variability, inter-animal variability may also contribute to the observed correlations (Figure 6). As such, the magnitude of pre-stimulus fluctuations could also be a factor in inter-animal variability in sensory-evoked hemodynamic responses. However to confirm it’s role in inter-trial variably we set each animals response magnitude between zero and unity. Furthermore, estimates of the size of the pre-stimulus fluctuations differed slightly depending on phase group (Figure 8). Phase group 3 (90–180°) was slightly (~18%) but significantly higher (one way ANOVA *F* = 3.680, Bonferroni corrected *post hoc* tests *p* < 0.05) than the other phase groups This slight difference is most likely due to the pre-stimulus period not being infinite in length. However, experiments were designed to maximize the number of trials that could be collected in each experimental subjects and as such the shortest pre-stimulus period was selected that would provide estimates of phase and size of fluctuations (Saka et al., 2010). Notwithstanding, to address these two possible caveats we re-analyzed data following normalizing each animals response magnitudes and size of pre-stimulus fluctuations between zero and unity for each phase group (Figure 9). Significant correlations between the size of the pre-stimulus fluctuation and the magnitude of the response were still apparent *t* in phase groups (1, 3, and 4) but not the group that was associated with the most modest correlation, phase group 2.

## Discussion

The present analyses demonstrate that both the size of pre-stimulus cortical hemodynamic fluctuations and their phase at stimulus onset may influence the magnitude of sensory-evoked hemodynamics responses. If the size of pre-stimulus cortical hemodynamics is examined alone, then a moderate positive correlation can be observed between the size of the pre-stimulus hemodynamic fluctuation for each trial and the magnitude of the evoked hemodynamic response. However, if trials are also sorted based on the phase of the pre-stimulus fluctuation it can be observed that the correlation between the size of the fluctuation and the subsequent sensory-evoked hemodynamic response varies depending on the phase of the fluctuation. For phases at stimulus onset where the ongoing fluctuation would have descended had a stimulus not been presented, there is a negative correlation between the size of the fluctuation and the subsequent sensory-evoked response. For other phases, where the ongoing fluctuation would have ascended had a stimulus not been presented, there is a positive correlation which varies in its degree depending on phase.

In contrast to the result of analyses presented here and in Saka et al. (2010) a recent conference report suggested that neither phase or magnitude of pre-stimulus BOLD fMRI fluctuations are related to the size of sensory-evoked hemodynamic signals (Herman et al., 2010). However, given the manner in which both phase of the pre-stimulus fluctuations influences whether the size of the fluctuation is positively or negatively correlated with the response size, it may be difficult to discern the association between the pre-stimulus fluctuation and the subsequent evoked response. Even if phase is examined in isolation, the relationship is not straightforward as the response magnitude increases in this order: group 1 (0–90°); group 2 (270–360°); group 3 (90–180°); and group 4 (180–270°). Consequently if investigators attempt to examine correlations between phase or size of pre-stimulus fluctuations and subsequent responses then it may be difficult to observe such associations even if they are present within the data set. Indeed, as different analysis methodology to that presented (Fox et al., 2006) also suggests that a mechanism associated with inter-trial variability in stimulus-evoked hemodynamics is a linear superposition of evoked and ongoing hemodynamics, then both phase and size of the pre-stimulus fluctuation would be expected to be associated with the magnitude of the subsequent evoked hemodynamic response.

Given the influence of ongoing hemodynamic fluctuations on the magnitude of sensory-evoked hemodynamic responses, the question arises as to whether individual trials of hemodynamics based imaging data are useful markers of inter-trial variability of evoked activity. The correlation co-efficient calculated in the present study corresponds to an *R*^2^ of 0.4, thus leaving a larger proportion of inter-trial variability in hemodynamics to be explained by sources other than pre-stimulus hemodynamic fluctuations. Sheth et al. (2003) measured neural and cortical total hemoglobin responses to sensory stimuli in an identical animal model to that used here. They found that for individual trials the relationship between whisker evoked activity and hemodynamics corresponded to a *R*^2^ = 0.36. Therefore, if the study of Sheth et al. (2003) and the present investigation are taken in tandem, it appears that ~80% of the variance in measured hemodynamic response magnitude could be explained. Given that each of the techniques will not be without some measurement error, this seems appropriate. The obvious limitation with the current and previous investigation (Saka et al., 2010) is that no measurements of neural activity were made. As such, the degree to which inter-trial variability in magnitudes of evoked activity and pre-stimulus hemodynamic fluctuations influence the magnitude of evoked hemodynamics in the same data set remains to be described. Furthermore, the factors influencing the phase and magnitude of pre-stimulus fluctuations also need to be elucidated. We have previously extensively discussed the possible neural and “vascular” origins of spontaneous hemodynamics (Saka et al., 2010). Notwithstanding, further work with combinations of electrophysiological and optical techniques (e.g., Boorman et al., 2010) will be required before inter-trial variability in hemodynamics is fully understood.

Nevertheless, a large proportion inter-trial variability is not to be related to evoked activity and thus, this seems at odds with reports that inter-trial variability in evoked fMRI responses reveals processing differences of cognitive relevance. However, if spontaneous ongoing hemodynamics are related to ongoing neural activity which has been demonstrated in several reports (Golanov et al., 1994; Logothetis et al., 2001; Shmuel and Leopold, 2008), then the phenomenon of linear summation between evoked and ongoing hemodynamics may suggest that the magnitude of the evoked fMRI response may also contain “information” not only about evoked activity but also prior ongoing activity. Given the temporal lag for hemodynamics to peak following both spontaneous ongoing and evoked activity it could be the case that hemodynamics at a single time point reflect neural activity for several preceding seconds. Indeed, several recent reports suggest that attributes of pre-stimulus neural activity as measured by EEG are predictive of the magnitude of subsequent evoked fMRI responses (Becker et al., 2011; Scheeringa et al., 2011). These studies demonstrated that both phase (Scheeringa et al., 2011) and power (Becker et al., 2011; Scheeringa et al., 2011) of the pre-stimulus ongoing EEG Alpha rhythm influenced subsequent fMRI responses. In the case of Alpha power, this influence on the magnitude of evoked fMRI responses, was due to a linear superposition of evoked and the aspects of spontaneous fMRI fluctuations that could be attributed to ongoing pre-stimulus alpha waves. The phase of alpha waves has also been found to be relevant to cognition such as whether subjects perceive a visual stimulus (Busch et al., 2009). Low frequency oscillations in neural activity that may be germane to spontaneous fluctuations in fMRI have also suggested to be important for perception (Monto et al., 2008), behavior, and even conscious awareness (VanRullen and Koch, 2003). For instance Monto et al. (2008) showed that the phase of low frequency EEG fluctuations could also determine whether or not subjects could detect a somatosensory stimuli. This has lead some authors to suggest that low frequency fluctuations may be away of selecting between sensory events (Schroeder and Lakatos, 2009).

The present study extends and confirms previous findings of a linear superposition of evoked and ongoing cortical hemodynamics (Fox et al., 2006; Saka et al., 2010) by suggesting that the size, in addition to the phase, of pre-stimulus hemodynamic fluctuations are associated with subsequent evoked response magnitude. A greater understanding of the relationships between evoked and ongoing activity, ongoing activity and spontaneous fluctuations in hemodynamics and fMRI signals, and evoked and ongoing fMRI signals are required to accurately interpret individual trials of fMRI data.

## Conflict of Interest Statement

The authors declare that the research was conducted in the absence of any commercial or financial relationships that could be construed as a potential conflict of interest.
